# *Deinococcus taeanensis* sp. nov., a Radiation-Resistant Bacterium Isolated from a Coastal Dune

**DOI:** 10.1007/s00284-022-03044-8

**Published:** 2022-09-25

**Authors:** Ji Hee Lee, Jong-Hyun Jung, Min-Kyu Kim, Sangyong Lim

**Affiliations:** 1 Division of Pathogen Resource Management, Korea Disease Control and Prevention Agency, Cheongju, 28160 Republic of Korea; 2 Radiation Research Division, Korea Atomic Energy Research Institute, Jeongeup, 56212 Republic of Korea; 3 Department of Radiation Science, University of Science and Technology, Daejeon, 34113 Republic of Korea

## Abstract

A Gram-stain-negative, nonspore-forming, nonmotile, aerobic, rod-shaped, and very pale orange-colored bacterial strain, designated TS293^T^, was isolated from a sand sample obtained from a coastal dune after exposure to 3kGy of gamma (γ)-radiation. Phylogenetic analysis based on the 16S rRNA gene sequences revealed that the isolate was a member of the genus *Deinococcus* and clustered with *D. deserti* VCD115^T^. The genome of strain TS293^T^ was 4.62 Mbp long (68.2% G + C content and 4124 predicted genes) divided into a 2.86Mb main chromosome and five plasmids. Many genes considered to be important to the γ-radiation and oxidative stress resistance of *Deinococcus* were conserved in TS293^T^, but genome features that could differentiate TS293^T^ from *D. deserti* and *D. radiodurans*, the type species of the *Deinococcus* genus, were also detected. Strain TS293^T^ showed resistance to γ-radiation with D_10_ values (i.e., the dose required to reduce the bacterial population by tenfold) of 3.1kGy. The predominant fatty acids of strain TS293^T^ were summed feature 3 (C_16:1_ *ω*6*c* and/or C_16:1_ *ω*7*c*) and iso-C_16:0_. The major polar lipids were two unidentified phosphoglycolipids and one unidentified glycolipid. The main respiratory quinone was menaquinone-8. Based on the phylogenetic, genomic, physiological, and chemotaxonomic characteristics, strain TS293^T^ represents a novel species, for which the name *Deinococcus taeanensis* sp. nov. is proposed. The type strain is TS293^T^ (= KCTC 43191^T^ = JCM 34027^T^).

**Supplementary Information:**

The online version contains supplementary material available at 10.1007/s00284-022-03044-8.

## Introduction

*Deinococcus* is one genus of three in the order *Deinococcales*, which is characterized by extreme ionizing radiation and desiccation resistance. It forms a monophyletic clade separated from the other two genera *Deinobacterium* and *Truepera*. Currently, the genus *Deinococcus* comprises 87 species with validly published names, whereas there is only one species in each genus *Deinobacterium* and *Truepera*, *Deinobacterium chartae,* and *Truepera radiovictrix*. Since *Deinococcus radiodurans* (*D. radiodurans*), originally named *Micrococcus radiodurans*, was first isolated from gamma (γ)-irradiated canned meat in 1956 [1], the members of the genus *Deinococcus* have been isolated from a wide range of natural and man-made environments, including soil [2], freshwater [3], air [4], and a car air-conditioning system [5]. These species have also been found in harsh environments, e.g., Antarctic soil [6], hot springs [7], arid land [8], and radiation-polluted soil [9]. Sample preparation using γ-irradiation treatment can also serve as a selective feature for the isolation of *Deinococcus* species [10, 11].

*D. radiodurans*, the type species of the genus, is an aerobic, Gram-positive, red-pigmented, nonsporulating, nonpathogenic bacterium [12]. The most unique characteristic of *D. radiodurans* is its extraordinary resistance to UV- and γ-radiation and oxidative stress, which makes it a promising research subject for DNA repair and antioxidant systems [12–14]. Investigation of the molecular mechanisms underlying the resistance phenotype common to all members of the genus *Deinococcus* can benefit from the availability of genomic information of various *Deinococcus* species. Hence, the genome sequence of *D. radiodurans* was published in 1999, genome sequencing of newly isolated *Deinococcus* species, such as *D. geothermalis* [15], *D. deserti* [16], *D. gobiensis* [17], *D. ficus* [18], and *D. terrestris* [2], and their comparative analyses have been performed to identify *Deinococcus*-specific proteins, or more specifically, unique DNA repair systems implicated in resistance. For instance, the metallopeptidase/repressor pair PprI (also called IrrE)/DdrO that controls the radiation/desiccation response (RDR) regulon is highly conserved across *Deinococcus* species [14, 19].

Our study sought novel bacteria from a sand sample collected from a coastal dune. A Gram-stain-negative, very pale orange-colored, and rod-shaped bacterial strain, designated TS293^T^ was isolated. Using a polyphasic approach, we established the taxonomic position of strain TS293^T^ in *Deinococcus* and analyzed its genomic features.

## Materials and Methods

### Isolation of Bacterial Strain and Culture Condition

Strain TS293^T^ was isolated from a sand sample obtained from a Taean coastal dune, Republic of Korea (GPS position; site 1 33° 21′ 44′′ N, 126° 32′ 00′′ E). Before isolation, the sand sample was irradiated by γ-radiation (3kGy). One gram of irradiated sand sample was mixed with saline solution and spread on tryptone glucose yeast agar (TGY; 5g tryptone, 3g yeast extract, 1g glucose, and 15g agar in 1l distilled water) using the standard dilution plating technique. After plating, plates were incubated at 30°C for 5 days. The very pale orange-colored isolate was routinely cultured on TGY and stored in glycerol (20%, w/v) at −70°C. Reference strains *D. arenae* KCTC 33741^T^ and *D. deserti* KACC 11782^T^ were purchased from the Korean collection for type cultures (KCTC) and the Korean agricultural culture collection (KACC), respectively.

### 16S rRNA Gene Sequencing and Phylogenetic Analysis

Bacterial DNA preparation and PCR amplification using universal primers 27F (5′-AGAGTTTGATCMTGGCTCAG-3′) and 1492R (5′-TACGGYTACCTTGTTACGACTT-3′) of the 16S rRNA gene were carried out as described previously [20]. The PCR product was sequenced by Macrogen Co., Ltd. (Republic of Korea). The 16S rRNA gene sequence similarities were calculated using the EzBioCloud server (www.ezbiocloud.net). Multiple alignments of the 16S rRNA gene sequences were performed using the CLUSTAL_W method [21] supplied by BioEdit version 7.2 software [22]. Phylogenetic trees (neighbor-joining [23], maximum-likelihood [24], and maximum-parsimony algorithms [25]) were performed using the software package MEGA version 7 [26]. Evolutionary distances of the neighbor-joining algorithm were computed using Kimura’s two-parameter model [27]. The robustness of the tree topology was evaluated by bootstrap analysis based on the 1000 resamplings [28].

### Genomic Analysis

For the whole-genome sequencing, the genomic DNA of strain TS293^T^ was extracted using a G-spin™ Genomic DNA Extraction Kit (iNtRON) following the manufacturer’s instructions. Whole-genome sequencing of the isolate was performed using PacBio RSII single-molecule real-time (SMRT) sequencing technology (Pacific Biosciences) at Macrogen Co., Ltd. De novo assembly was performed using the hierarchical genome assembly process version 3 (HGAP3) [29]. After the whole genome was assembled, genes were identified and annotated by Prokka pipeline version 1.13 [30]. Gene functions were then annotated using the eggNOG database [31]. The DNA G + C content was calculated directly from the genome sequence. The average nucleotide identity (ANI) between a given pair of genomes was determined by using the JSpecies software based on the BLAST algorithm [32, 33]. The distance matrix based on the ANI values obtained was used in MEGA 7 software to perform a genome-scale phylogenetic analysis [26].

### γ-Radiation-Resistant Analysis

To determine the survival rate after exposure to γ-radiation, strain TS293^T^, *Deinococcus arenae,* and *D. radiodurans* R1^T^ (positive control) were grown to an early stationary phase and irradiated at room temperature using a ^60^Co-gamma irradiator (AECL, IR-79; MDS Nordion International Co., Ltd.) with doses of 3, 6, 9, 12kGy at the Advanced Radiation Technology Institute in the Republic of Korea. Following irradiation, the strains were serially diluted tenfold and then spotted on TGY agar plates in triplicate. The plates were incubated at 30°C for 3 days. The number of colony-forming units (CFU) of strains was determined, and then the survival rate was calculated.

### Phenotypic and Biochemical Characterization

Growth on various standard bacteriological media was tested using TGY, R2A agar (MB cell), nutrient agar (NA; Difco), tryptic soy agar (TSA; Difco), and Luria–Bertani agar (LB; MB cell). Growth temperature (at 4, 10, 15, 20, 25, 30, 37, 40, or 45°C) was tested on TGY agar. The pH range for growth was determined in TGY broth adjusted to pH 4–11 (at 1 pH intervals) using 100mM acetate buffer (pH 4–5), 100mM MES (pH 6), 100mM HEPES (pH 7–8), 100mM CHES (pH9-10), and 100mM CAPS (pH 11). The requirement and tolerance to NaCl [final concentration: 0, 0.5, 1, 2, 3, 4, or 5% (w/v)] for growth was tested on TGY broth. Anaerobic growth was tested on TGY agar in a jar containing AnaeroGen (Thermo Scientific), for up to 14 days at 30°C. Cell morphology was observed by transmission electron (Tecnai 12, FEI) microscopy. Cell motility was investigated with 0.3% semi-solid TGY agar, and gliding motility was assessed by examining wet mounts of a 48h TGY broth culture under phase-contrast microscopy (ICC50, Leica). The Gram reaction was determined using the Gram staining method and the KOH method [34]. Catalase and oxidase activities were determined using 3% (v/v) hydrogen peroxide and 1% (w/v) tetramethyl-*p*-phenylenediamine, respectively. Biochemical tests, enzyme activities, and utilization of carbohydrates were evaluated using the API 20NE and API ZYM kits (bioMérieux) following the manufacturer’s instructions.

### Chemotaxonomic Characterization

For analysis of the cellular fatty acid composition, strain TS293^T^ and reference strains were grown on TGY agar for 3 days at 30°C. Extraction of fatty acid methyl esters (FAME) and separation by gas chromatography (GC) were performed using the Instant FAME method of the Microbial Identification System (MIDI) version 6.1 and the TSBA6 database [35]. To analyze polar lipids and isoprenoid quinone, cells of strain TS293^T^ grown in R2A broth for 3 days at 30°C were harvested and freeze dried. Polar lipids were extracted using standard procedures. Extracted polar lipids were separated by two-dimensional thin-layer chromatography (TLC) using TLC silica gel 60F254 (Merck). Chromatograms were developed in the first dimension with a mixture of chloroform/methanol/water (65:25:4 by volume) and in the second dimension with chloroform/acetic acid/methanol/water (80:18:12:5 by volume) [36]. Isoprenoid quinones were extracted and analyzed by high-performance liquid chromatography (HPLC) [37].

## Results and Discussion

### 16S rRNA Phylogenetic Analysis

**Fig. 1:**
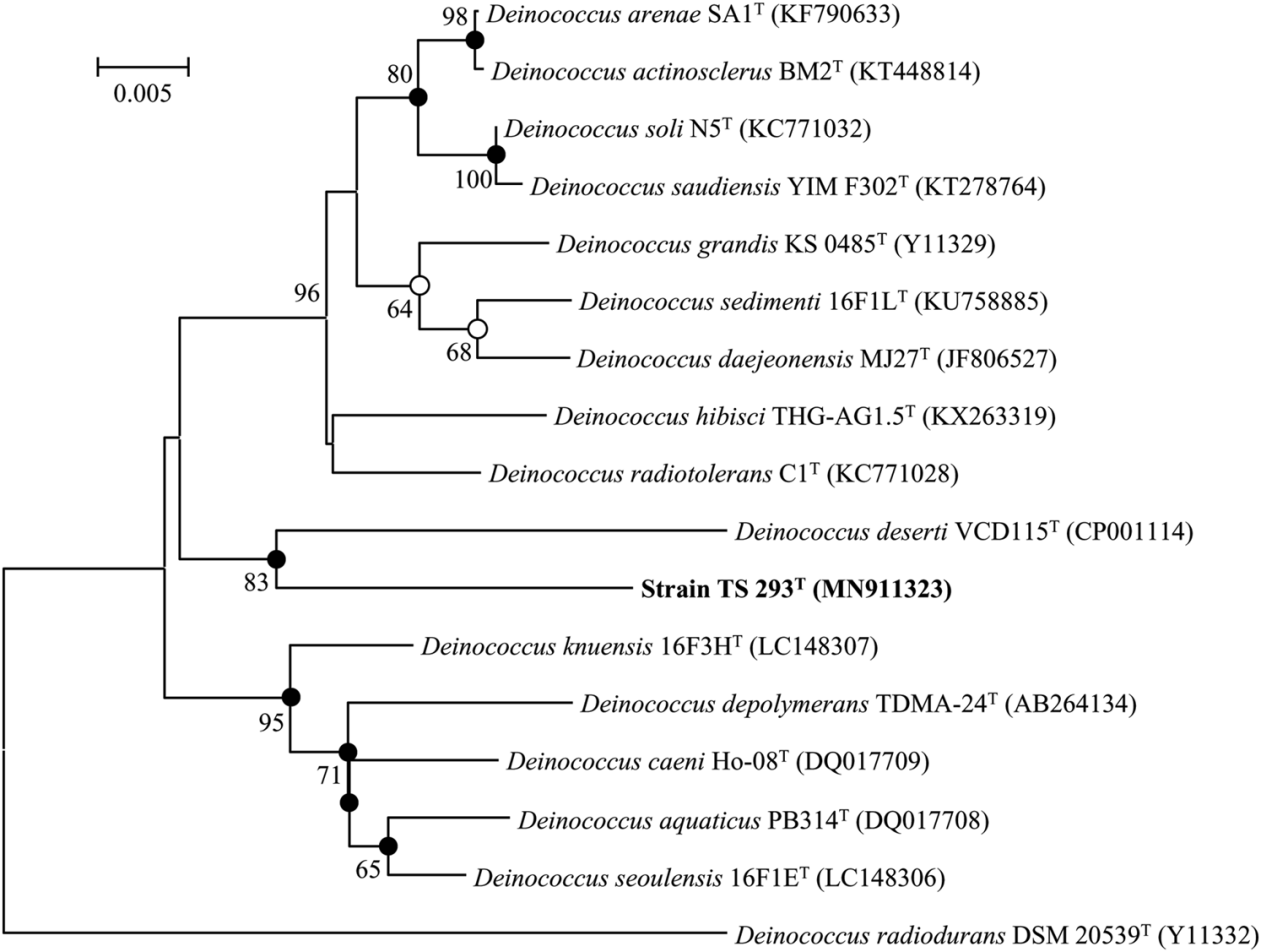
A neighbor-joining tree based on the16S rRNA gene sequences showing the phylogenetic position of strain TS293^T^ and related strains of the genus *Deinococcus.* Evolutionary distances, generated using the Kimura two-parameter model, are based on the 1371 unambiguously aligned nucleotides. Bootstrap values greater than 60% (1000 resamplings) for nodes conserved among neighbor-joining analyses are shown. Closed circles indicate that the corresponding nodes were also recovered in trees generated with the maximum-parsimony and maximum-likelihood algorithms. Opened circles indicate branches of the tree that were also recovered using the maximum-likelihood algorithm. *Deinococcus radiodurans* DSM 20539^ T^ (Y11332) was used as an outgroup. Bar, 0.005 substitutions per nucleotide position

The 16S rRNA gene sequence (1433bp) of strain TS293^T^ was obtained (GenBank accession no. MN911323). The sequence comparison using the EzBioCloud server indicated that our isolate was closely related to members of the genus *Deinococcus*. Strain TS293^T^ showed the highest 16S rRNA gene sequence similarity to *D. arenae* SA1^T^ (96.0%). Sequence similarity with other members of the genus *Deinococcus* was less than 96.0%. The neighbor-joining phylogenetic tree (Fig. 1) showed that strain TS293^T^ formed a distinct branch within the *Deinococcus*. Strain TS293^T^ clustered with *D. deserti* VCD115^T^, supported with an 83% bootstrap value. This two-strain cluster was also observed in maximum-likelihood and maximum-parsimony algorithm trees (Fig. S1). The phylogenetic analysis indicated strain TS293^T^ represents a novel species within the genus *Deinococcus*.

### Genomic Analysis

The genome of TS293^T^ is composed of 6 replicons: a 2.86Mb main chromosome and five plasmids, whose range in size is from 447 to 79kb (Table 1). The total genomic G + C content of strain TS293^T^ was 68.2mol% (Table 1), which was within the range of G + C levels (62–70mol%) of *Deinococcus* [38]. The total length of the TS293^T^ genome was 4,618,413bp and was larger than those of the two species *D. arenae* (GenBank accession no. NZ_BMQG00000000; 4,215,994bp) and *D. deserti* (GenBank accession no. NC_012526–NC_012528; 3,855,329bp). A whole-genome-based phylogenetic tree was generated by ANI pairwise comparisons between the complete and draft genome sequences available for 13 *Deinococcus* species of the 16 species presented in Fig. 1. Of note, despite the high 16S rRNA gene sequence similarity, TS293^T^ and *D. arenae* did not cluster in the same clade (Fig. S2), which is consistent with the previously determined phylogenies (Fig. 1). The ANI values between TS293^T^ and the two species *D. radiodurans* and *D. deserti* were 74.2% and 74.4%, respectively, which are much lower than the threshold of species delineation of 95% ANI [32], suggesting that TS293^T^ can represent distinct species.

**Table 1:** General characteristics of the TS293^T^ genome

Molecule	Chromosome	Plasmids					All
		P1	P2	P3	P4	P5	
Size (bp)	2,855,189	446,704	430,426	421,178	386,169	78,747	4,618,413
GC content (%)	68.7	69.1	65.8	69.1	65.7	62.1	68.2
Coding density (%)							
Protein-coding genes	2646	365	380	341	331	61	4124
Pseudogenes	41	16	20	10	22	1	110
tRNAs	49	1	-	-	-	-	50
rRNAs	9	3	-	-	-	-	12
ncRNA	2	1	-	-	-	-	3
GenBank accession number	CP083455	CP083456	CP083457	CP083458	CP083459	CP083460	

The genome of strain TS293^T^ contained 4124 protein-coding sequences (CDSs), 50 tRNA genes, and 12 rRNA genes (4 copies each of 5S, 16S, 23S). Of 4124 protein-coding genes, 3881 genes were assigned to the COG of proteins (Table S1). In the COG category assignment, except for poorly characterized categories (R and S), amino acid transport and metabolism (E), replication, recombination, and repair (L), and carbohydrate transport and metabolism (G) showed high abundance (Table S1). When compared with *D. deserti*, TS293^T^ showed a higher ratio (7.4%) of genes in the COG category L (Table S1). Remarkably, TS293^T^ contained 148 complete and partial mobile genetic elements, such as transposase.

We analyzed the DNA repair, antioxidant, and *Deinococcus*-specific Ddr and Ppr proteins in TS293^T^ and compared them with those of the closely related species *D. deserti* and the type species of this genus, *D. radiodurans*. Most of the proteins analyzed here were well conserved in the three *Deinococcus* species (Tables S2 to S4). However, the fusion protein of AdaA and AlkA, which play protective roles against DNA alkylating agents, was detected only in TS293^T^, showing the difference between TS293^T^, *D. radiodurans* and *D. deserti* (Table S2). This difference was also observed in antioxidant proteins. The Cu/Zn-containing superoxide dismutase SodC and the Mn-containing catalase MnCat were absent and present, respectively, only in TS293^T^ (Table S3). It is worth to noting that the recombinational repair-related protein RecA, the bacterioferritin comigratory protein Bcp, and the alkyl hydroperoxidase D (AhpD)-like protein YciW were present in all of the three species, but in different numbers (Tables S2 and S3).

On the whole, the protein profile of TS293^T^ was more similar to that of *D. deserti* than *D. radiodurans*. Some proteins, such as Udg4 (uracil−DNA glycosylase) and YhDJ (DNA modification methylase), present in *D. radiodurans* were absent in both TS293^T^ and *D. deserti*, and the spore photoproduct lyase SplB that repairs crosslinked thymine bases caused by UV radiation and the UvrD-like helicase were detected in the two species (Table S2). The RDR regulon is controlled by PprI and DdrO which are highly conserved in *Deinococcus*.

**Fig.2:**
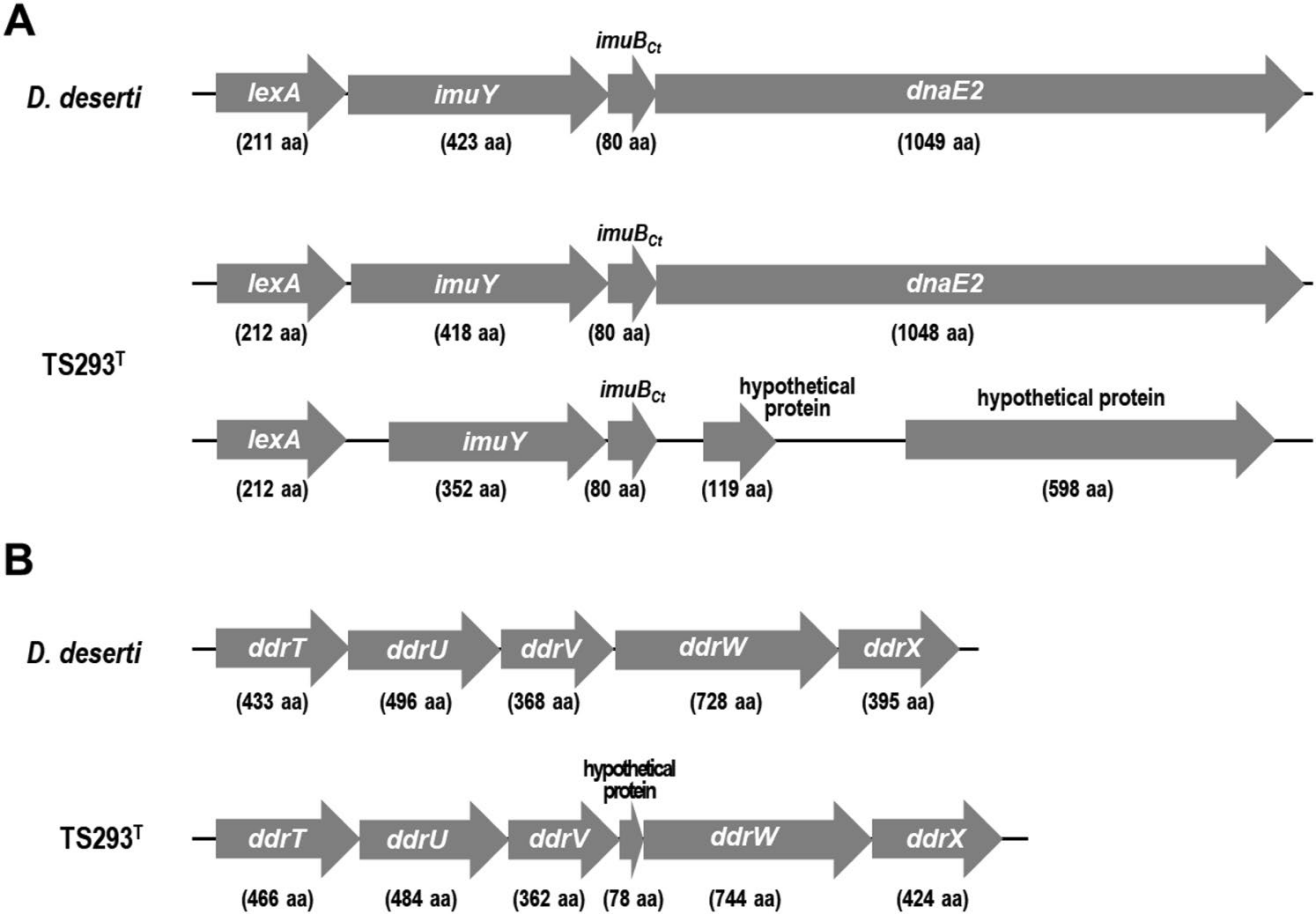
Gene arrangement of the *lexA*-*imuY*-*imuB*_*Ct*_-*dnaE2* **(A)** and *ddrTUVWX* operons **(B)** in *D. deserti* and TS293^T^

Recently, it has been reported that several *Deinococcus* species possess not only the PprI/DdrO system but also an SOS-dependent pathway to induce DNA repair genes, in which activated RecA stimulates the autocleavage of LexA, the repressor of the SOS regulon [19]. One of the *Deinococcus* SOS regulons found in *D. deserti* is the *lexA*-*imuY*-*imuB*_*Ct*_-*dnaE2* operon coding for error-prone translesion polymerase DnaE2 and two other auxiliary proteins, ImuY and the C-terminal domain of ImuB protein (ImuB_Ct_) [19]. Although *D. deserti* possesses the complete *lexA*-*imuY*-*imuB*_*Ct*_-*dnaE2* mutagenesis cassette, TS293^T^ had the complete one and the partial one without *dnaE2* (Fig. 2A). The five-gene operon *ddrTUVWX*, one of the new RDR members found in *D. deserti* [39], was also present in TS293^T^ (Table S4). However, another gene encoding a hypothetical protein of 78 amino acid residues was present between *ddrV* and *ddrW* in TS293^T^ (Fig. 2B). These results indicate a high level of genetic differentiation between these two *Deinococcus* species.

### γ-Radiation-Resistant Analysis

**Fig. 3:**
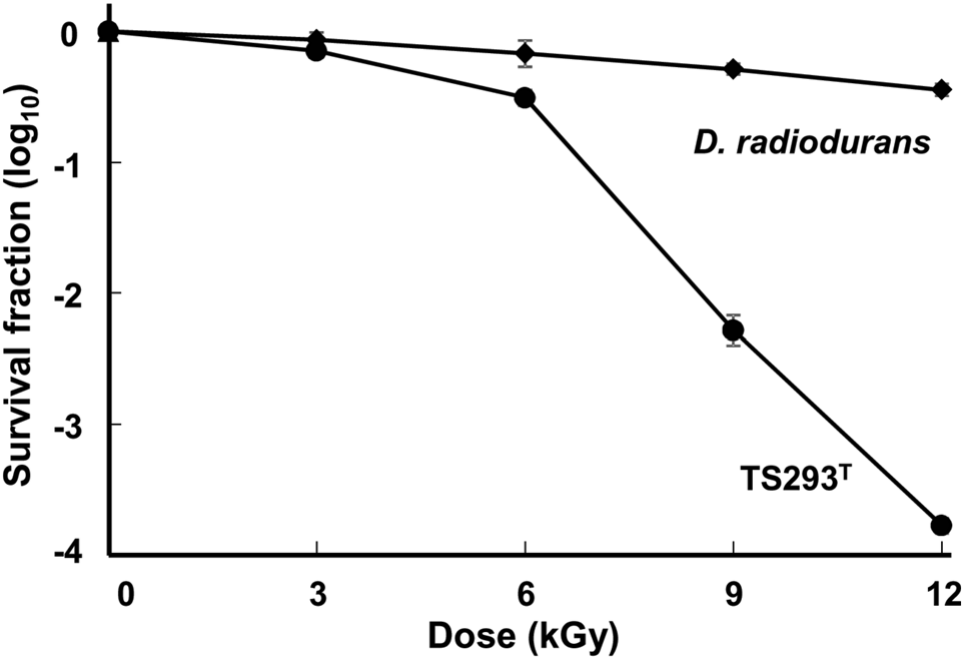
Representative survival curves of strain TS293^T^ after treatment with various doses of γ-radiation. *Deinococcus radiodurans* R1^T^ and *Escherichia coli* MG1655.^T^ were used as positive and negative controls, respectively. The error bars represent the standard deviations of three independent experiments (*n* = 3)

After exposure to 3, 6, and 9kGy γ-radiation, strain TS293^T^ showed 72.2, 31.7, and 0.6% cell survival, respectively, yielding a D_10_ of 3.1kGy (Fig. 3). Because *D. deserti* KACC 11782^T^ was known to have a D_10_ value of > 10kGy [40], TS293^T^ was less resistant to γ-radiation than *D. deserti*. At 3kGy, *Escherichia coli* were reduced to ~ 2 log CFU/ml in this study (data not shown).

### Phenotypic and Biochemical Characterization

Cells were observed to be Gram-stain-negative, nonspore forming, nonmotile, aerobic, and rod shaped (1.2–1.4 × 1.5–2.8μm in size). Most of the *Deinococcus* species are Gram stain positive, but some are Gram stain negative [40]. Strain TS293^T^ grew on TGY, NA, and R2A but not on TSA or LB. The strain was able to grow with 0–0.5% (w/v) NaCl, at pH 6–8 (optimally at pH 7–8) and 15–37°C (optimally at 30°C). Colonies were observed to be circular, smooth, very pale orange colored, and 1–3mm in diameter after incubation on TGY agar for 3 days. The strain was found to be positive for catalase, but negative for oxidase. Esculin was hydrolyzed, but arginine, urea, and gelatin were not. The other results of the physiological and biochemical analyses are given in the description and Table 2. There are several phenotypic characteristics such as enzyme activity of alkaline phosphatase, naphthol-AS-BI-phosphohydrolase, and *β*-glucosidase, and no assimilation of mannitol that differentiate strain TS293^T^ and *D. deserti*.

**Table 2:** Phenotypic characteristics that differentiate strain TS293^T^ from related *Deinococcus* species

	TS293T	*D. deserti*
Colony color	Very pale orange	Whitish
Growth at		
LB	–	–
TSA	–	+
Reduction of nitrate to nitrite	–	–
Hydrolysis of gelatin	–	–
Enzyme activity (API ZYM):		
Alkaline phosphatase	+	–
α-Chymotrypsin	+	+
Cystine arylamidase	+	+
β-Galactosidase	+	–
β-Glucosidase	+	–
Naphthol-AS-BI-phosphohydrolase	+	–
Trypsin	+	+
Valine arylamidase	+	+
Assimilation (API 20NE):		
Malate	–	–
Maltose	–	–
Mannitol	–	+
Mannose	–	–
DNA G + C content (mol%)*	68.2	63

These data were from this study. + , positive; –, negative. Both strains were positive for aerobic metabolism, catalase activity, hydrolysis of esculin, and the enzyme activity of acid phosphatase, esterase (C4), esterase lipase (C8), *α*-glucosidase, and leucine arylamidase. Both strains were negative for Gram reaction, reduction of nitrate to nitrogen, indole production, oxidase activity, hydrolysis of arginine, and urea, the enzyme activity of *α*-fucosidase, *α*-galactosidase, *β*-glucuronidase, *N*-acetyl-*β*-glucosaminidase, and *α*-mannosidase, and assimilation of adipate, arabinose, caprate, citrate, gluconate, *N*-acetyl-glucosamine, and phenyl-acetate

^a^Data are from the genome sequences of GenBank

### Chemotaxonomic Characterization

The predominant fatty acids (> 5.0% of total fatty acids) of strain TS293^T^ were summed feature 3 (C_16:1_ *ω*6*c* and/or C_16:1_ *ω*7*c*) (36.2%), iso-C_16:0_ (21.7%), C_16:0_ (8.3%), and C_15:1_ *ω*6*c* (6.0%) (Table S5). Summed feature 3 (C_16:1_ *ω*6*c* and/or C_16:1_ *ω*7*c*) and C_16:0_ were presented as major fatty acids in strain TS293^T^ and *D. deserti*. However, strain TS293^T^ contained a higher proportion of iso-C_16:0_ when compared with *D. deserti*, and C_16:1_ *ω*9*c* detected in *D. deserti* was absent in TS293^T^. The predominant polar lipid of strain TS293^T^ were two unidentified phosphoglycolipids (PGL1, PGL2), and one unidentified glycolipid (GL6) (Fig. S3). The main respiratory quinone of strain TS293^T^ was menaquinone-8. These support the affiliation to the genus *Deinococcus* [40].

### Taxonomic Conclusion

The genotypic, phenotypic, chemotaxonomic, and γ-radiation-resistant analyses presented in this study clearly show that the strain differs from the related species *D. deserti* analyzed here. The physiological characteristics of strain TS293^T^ and *D. deserti* are summarized in Table 1. In conclusion, we suggest that strain TS293^T^ represents a novel species of the genus *Deinococcus,* for which the name *Deinococcus taeanensis* sp. nov. is proposed.

### Description of *Deinococcus taeanensis* sp. nov.

*Deinococcus taeanensis* sp. nov. (tae-an-en’-sis. N.L. masc. adj. *taeanensis*: of or belonging to Taean, Republic of Korea, the geographical origin of the type strain of the species.)

Cells are Gram stain negative, nonspore forming, nonmotile, aerobic, and rod shaped, approximately 1.2–1.4μm in diameter and 1.5–2.8μm in length. Colonies are observed to be circular, smooth, very pale orange colored, and 1–3mm in diameter after incubation on TGY agar for 3 days. Growth occurs on TGY, NA and R2A, with 0–0.5% (w/v) NaCl (optimally 0%), at pH 6–8 (optimally pH 7–8) and at 15–37°C (optimally 30°C). Strain TS293^T^ tolerated γ-radiation with a D_10_ value of 3.1kGy and was positive for catalase, but negative for oxidase. Cells are positive for hydrolysis of esculin, assimilation of glucose, the enzyme activity of alkaline phosphatase, esterase (C4), esterase lipase (C8), leucine arylamidase, valine arylamidase, cystine arylamidase, trypsin, *α*-chymotrypsin, acid phosphatase, naphthol-AS-BI-phosphohydrolase, *β*-galactosidase, *α*-glucosidase, and *β*-glucosidase. The predominant fatty acids are summed feature 3 (C_16:1_ *ω*6*c* and/or C_16:1_ *ω*7*c*) and iso-C_16:0_. The major polar lipids are two unidentified phosphoglycolipids and one unidentified glycolipid, and the main respiratory quinone is menaquinone-8. Its genome is 4.6Mb with a DNA G + C content of 68.2mol%, which contained 4,124 CDSs.

The type strain is TS293^T^ (= KCTC 43191^T^ = JCM 34027^T^), isolated from sand in the Republic of Korea.

The GenBank accession number for the 16S rRNA gene sequence and the genome sequence of strain TS293T are MN911323 and CP083455–CP083460, respectively.

## Supplementary Information

Below is the link to the electronic supplementary material.

Supplementary file1 (PDF 597 KB)

## Data Availability

The GenBank/EMBL/DDBJ accession number for the 16S rRNA gene sequence and whole-genome sequence of strain TS293^T^ are MN911323 and CP083455–CP083460, respectively.
