# Supplementary material for: Deinococcus taeanensis sp. nov., a Radiation-Resistant Bacterium Isolated from a Coastal Dune
Source: Curr Microbiol. 2022 Sep 25;79(11):334. doi: 10.1007/s00284-022-03044-8 (PMC9510100; doi:10.1007/s00284-022-03044-8)
Supplement: Supplementary file 1 — Supplementary file1 (PDF 597 KB) [file 284_2022_3044_MOESM1_ESM.pdf]

Supplementary Information

Deinococcus taeanensis sp. nov., a radiation-resistant bacterium isolated from coastal dune

Current Microbiology

Ji Hee Lee[1], Jong-Hyun Jung[2], Min-Kyu Kim[2] and Sangyong Lim[2,3]*

[1]Division of Pathogen Resource Management, Korea Disease Control and Prevention Agency, Cheongju 28160, Republic of Korea, [2]Radiation Research Division, Korea Atomic Energy Research Institute, Jeongeup, 56212, Republic of Korea, [3]Department of Radiation Science, University of Science and Technology, Daejeon 34113, Republic of Korea.

***Corresponding author: Sangyong Lim (Ph.D.),** E-mail: saylim@kaeri.re.kr

Supplementary Table 1. COG functional categories of *D. deserti* (Ddes) and TS293^T

Category	Name	TS293^T		Ddes	
		Count	Ratio (%)	Count	Ratio (%)
**Information storage and processing**					
J	Translation, ribosomal structure, and biogenesis	166	4.22	160	5.54
A	RNA processing and modification	0	0	0	0
K	Transcription	203	5.16	164	5.68
L	Replication, recombination, and repair	291	7.40	149	5.16
B	Chromatin structure and dynamics	2	0.05	2	0.07
**Cellular processes and signaling**					
D	Cell cycle control, cell division, chromosome partitioning	32	0.81	26	0.90
Y	Nuclear structure	0	0	0	0
V	Defense mechanisms	54	1.37	35	1.21
T	Signal transduction mechanisms	226	5.75	175	6.06
M	Cell wall/membrane/envelope biogenesis	126	3.21	109	3.77
N	Cell motility	8	0.20	6	0.21
Z	Cytoskeleton	0	0	0	0
W	Extracellular structures	1	0.03	0	0
U	Intracellular trafficking, secretion, and vesicular transport	35	0.89	32	1.11
O	Posttranslational modification, protein turnover, chaperones	130	3.31	119	4.12
**Metabolism**					
C	Energy production and conversion	169	4.30	146	5.05
G	Carbohydrate transport and metabolism	260	6.61	210	7.27
E	Amino acid transport and metabolism	316	8.04	288	9.97
F	Nucleotide transport and metabolism	87	2.21	80	2.77
H	Coenzyme transport and metabolism	103	2.62	104	3.60
I	Lipid transport and metabolism	93	2.37	82	2.84
P	Inorganic ion transport and metabolism	181	4.60	149	5.16
Q	Secondary metabolites biosynthesis, transport, and catabolism	57	1.45	57	1.97
**Poorly characterized**					
R	General function prediction only	375	9.54	0	0
S	Function unknown	1016	25.85	796	27.55

Supplementary Table 2. DNA repair proteins in *D. radiodurans* (Drad), *D. deserti* (Ddes), and TS293^T

Common name	COG / Domain information	Description	Drad	Ddes	TS293^T
**Base excision repair**					
AlkA	COG0122	3-methyladenine DNA glycosylase/8-oxoguanine DNA glycosylase	1	1	1
Mpg	COG2094	3-methyladenine DNA glycosylase	1	1	1
Ung	COG0692	Uracil DNA glycosylase	1	1	1
Udg4	COG1573	Uracil-DNA glycosylase	1	0	0
Mug	COG3663	G:T/U-mismatch repair DNA glycosylase	1	1	1
Nfi	COG1515	Deoxyinosine 3'endonuclease (endonuclease V)	1	0	1
MutY	COG1194	Adenine-specific DNA glycosylase, acts on AG and A-oxoG pairs	1	1	1
MutM (Fpg)	COG0266	Formamidopyrimidine-DNA glycosylase	1	1	1
Nth	COG0177	Endonuclease III	3	3	3
XthA	COG0708	Exonuclease III	1	2	2
**Mismatch repair**					
MutL	COG0323	DNA mismatch repair ATPase	1	1	1
MutS	COG0249	DNA mismatch repair ATPase	1	1	1
MutS2	COG1193	dsDNA-specific endonuclease/ATPase	1	1	1
MutH	COG3066	DNA mismatch repair protein MutH	0	0	0
Vsr	COG3727	very short path repair endonuclease (G:T-mismatch)	0	0	0
YcjD	COG2852	endonuclease domain-containing protein (DUF559)	1	0	0
Dcm	COG0270	Site-specific DNA-cytosine methylase	0	0	0
Dam	COG0338	Site-specific DNA-adenine methylase	0	0	0
YhdJ	COG0863	DNA modification methylase	1	0	0
**Direct reversal**					
AdaA	COG2169	Bifunctional transcriptional activator/DNA repair enzyme	0	0	0
AdaA/AlkA	COG2169/COG0122	fusion AdaA/AlkA	0	0	1
PhrB	COG0415	Deoxyribodipyrimidine photolyase	0	0	0
SplB	COG1533	Spore photoproduct lyase family protein	0	1	1
AdaB	COG0350	O6-methylguanine-DNA-protein-cysteine methyltransferase	0	0	0
Dut	COG0756	dUTPase	0	0	0
Dcd	COG0717	Deoxycytidine triphosphate deaminase	0	1	1
AlkB	COG3145	Alkylated DNA repair dioxygenase	0	0	0
RdgB	COG0127	Inosine/xanthosine triphosphate pyrophosphatase	1	1	1

DJ-1/PfpI family	COG0693	methylglyoxal and glyoxal deglycase	2	1	2
**Nucleotide excision repair**					
UvrA	COG0178	Excinuclease UvrABC, ATPase subunit	1	1	1
UvrA2	COG0178	Excinuclease UvrABC, ATPase subunit	1	1	1
UvrB	COG0556	Excinuclease UvrABC, helicase subunit	1	1	1
UvrC	COG0322	Excinuclease UvrABC, nuclease subunit	1	1	1
UvrD	COG0210	Superfamily I DNA or RNA helicase	1	1	1
UvrD-like	COG0210	Superfamily I DNA or RNA helicase	0	1	1
SSL2 (Rad25)	COG1061	Superfamily II DNA or RNA helicase (multi-form)	1	1	2
Mfd	COG1197	Transcription-repair coupling factor (superfamily II helicase)	1	1	1
Uve (UvsE)	COG4294	UV DNA damage repair endonuclease	1	1	1
Atl1	COG3695	Alkylated DNA nucleotide flippase Atl1, Ada-like DNA-binding domain	1	1	1
**Recombinational repair**					
RecA	COG0468	Protein RecA; Recombinase A	1	3	2
RecF	COG1195	Recombinational DNA repair ATPase RecF	1	1	1
RecO	COG1381	DNA repair protein RecO; Recombination protein O	1	1	1
RecR	COG0353	Recombinational DNA repair protein RecR	1	1	1
RecJ	COG0608	Single-stranded DNA-specific exonuclease	1	1	1
RecN	COG0497	DNA repair ATPase RecN	1	1	1
RecQ	COG0514	Superfamily II DNA helicase RecQ (including HRDC domains)	1	1	1
RecQ-like	COG0514	RecQ-like (without RQC and HRDC domains)	0	1	1
HRDC domain		HRDC-domain containing protein	1	1	1
RecD	COG0507	ATP-dependent RecD-like DNA helicase (RecD2)	1	1	1
SbcC	COG0419	DNA repair exonuclease SbcCD, ATPase subunit	1	1	1
SbcD	COG0420	DNA repair exonuclease SbcCD, nuclease subunit	1	1	1
RuvA	COG0632	Holliday junction resolvasome RuvABC, DNA-binding subunit	1	1	1
RuvB	COG2255	Holliday junction resolvasome RuvABC, DNA helicase subunit	1	1	1
RuvC	COG0817	Holliday junction resolvasome RuvABC, endonuclease subunit	1	1	1
RecG	COG1200	RecG-like helicase	1	1	1
RecB	COG1074	ATP-dependent exoDNAse (exonuclease V) beta subunit	0	0	0
RecC	COG1330	Exonuclease V gamma subunit	0	0	0
RecT	COG3723	Recombinational DNA repair protein RecT	0	0	0
RecX	COG2137	SOS response regulatory protein OraA/RecX, interacts with RecA	1	1	1

Supplementary Table 3. Antioxidant proteins in *D. radiodurans* (Drad), *D. deserti* (Ddes), and TS293^{T}

Common name	COG/ Domain information	Description	Drad	Ddes	TS-293^{T}
**Superoxide dismutases (SODs)**					
SodA	COG0605	Mn-containing SOD	1	1	1
SodB	COG0605	Fe-containing SOD	0	0	0
SodC	COG2032	Cu/Zn-containing SOD	2	2	0
**Catalases**					
KatE	COG0753	monofunctional heme catalase	2	1	1
KatG	COG0376	bifunctional heme catalase-peroxidase	0	0	0
MnCat	COG3546	(non-heme) Mn-containing catalase	0	0	1
**Peroxiredoxins (Prxs) and Prx-related proteins**					
Bcp	cd03017	bacterioferritin comigratory protein	3	2	4
Tpx	COG2077	thiol peroxidase	0	0	0
AhpF	COG3634	alkyl hydroperoxide reductase subunit AhpF	0	0	0
AhpE	cd03018/cd02971	atypical type of AhpC	1	1	1
AhpD	COG2128	alkyl hydroperoxidase D-like protein (YciW)	1	2	4
**Thioredoxins (Trxs) and Trx-related proteins**					
TrxA	cd02947	thioredoxin	1	1	1
TrxC	cd02947	thioredoxin	1	1	1
TrxR	IPR005982	thioredoxin reductase	1	1	1
**Glutaredoxins (Grxs)**					
GrxC	COG0695	glutaredoxin	4	4	3
GrxB	COG2999	glutaredoxin 2	0	0	0
GrxD	COG0278	glutaredoxin-related protein	0	0	0
**Bacillithiol (BSH)**					
BshA		BSH biosynthesis glycosyltransferase	1	1	1
BshB1		BSH biosynthesis deacetylase	1	1	1
BshC		BSH biosynthesis cysteine-adding enzyme	1	1	1
YpdA		BSH reductase	1	1	1
YtxJ		bacilliredoxin (Brx)	1	1	1

Supplementary Table 4. *Deinococcus*-specific Ddr and Ppr proteins in *D. radiodurans* (Drad), *D. deserti* (Ddes), and TS293^T

Name	Description	Drad	Ddes	TS293^T	Note
**DNA damage response (Ddr) proteins**					
DdrA	single-stranded DNA-binding protein	1	1	1	
DdrB	single-stranded DNA-binding protein	1	1	1	
DdrC	DNA damage response protein	1	1	1	
DdrD	multidrug DMT transporter	1	1	1	
DdrE	zinc metallopeptidase	1	1	1	
DdrF	hypothetical protein	1	0	0	detected only in *D. radiodurans*
DdrG	hypothetical protein	1	0	0	
DdrH	hypothetical protein	1	1	1	
DdrI	CRP/FNR family transcriptional regulator	1	1	1	
DdrJ	NADAR family protein	1	0	1	
DdrK					wrong annotation
DdrL	heme-binding domain-containing protein	1	0	0	
DdrM	cation-translocating P-type ATPase	1	0	1	
DdrN	GNAT family N-acetyltransferase	1	1	1	
DdrO	transcriptional regulator, XRE family	1	2	2	
DdrP	RNA ligase family protein	1	0	1	
DdrQ	hypothetical protein	0	1	1	
DdrR	damage-inducible protein DinB	1	1	1	
DdrS	hypothetical protein	0	1	1	
DdrT	SWIM zinc finger family protein	0	1	1	
DdrU	DUF5691 domain-containing protein	0	1	1	
DdrV	AAA family ATPase	0	1	1	
DdrW	DUF5682 family protein	0	1	1	
DdrX	VWA domain-containing protein	0	1	1	
**Pleiotropic protein promoting DNA repair (Ppr) proteins**					
PprA	DNA repair protein PprA	1	1	2	
PprI	ImmA/IrrE family metallo-endopeptidase	1	1	1	
PprM	cold shock domain-containing protein	1	2	2	

Supplementary Table 5. Fatty acid composition of strain TS293^T and *Deinococcus deserti*

	**TS293^T**	**D. deserti**
Saturated		
$C_{14:0}$	Tr	Tr
$C_{15:0}$	3.6	1.8
$C_{16:0}$	8.3	10.9
$C_{17:0}$	3.6	2.7
Unsaturated		
$C_{15:1}$ *ω6c*	6.0	2.2
$C_{16:1}$ *ω5c*	1.5	Tr
$C_{16:1}$ *ω9c*	ND	3.9
$C_{17:1}$ *ω6c*	3.9	2.8
$C_{17:1}$ *ω8c*	1.2	7.4
Branched-chain fatty acid		
iso-$C_{15:0}$	2.0	Tr
iso-$C_{16:0}$	21.7	3.9
iso-$C_{16:1}$ H	3.8	2.1
iso-$C_{17:0}$	2.8	2.7
iso-$C_{18:0}$	Tr	Tr
iso-$C_{18:1}$ H	Tr	1.6
Summed feature†		
3	36.2	41.6
4	Tr	Tr
8	Tr	1.8
9	Tr	8.5

All data from this study. –, Not detected; Tr, trace (<1%).

†Summed features represent two or three fatty acids that cannot be separated by the Microbial Identification System. Summed feature 3 comprises $C_{16:1}$ *ω6c* and/or $C_{16:1}$ *ω7c*; summed feature 4 comprises iso-$C_{17:1}$ I and/or anteiso-$C_{17:1}$ B; summed feature 8 comprises $C_{18:1}$ *ω6c* and/or $C_{18:1}$ *ω7c*; summed feature 9 comprises iso-$C_{17:1}$ *ω6c* and/or 10-methyl $C_{16:0}$.

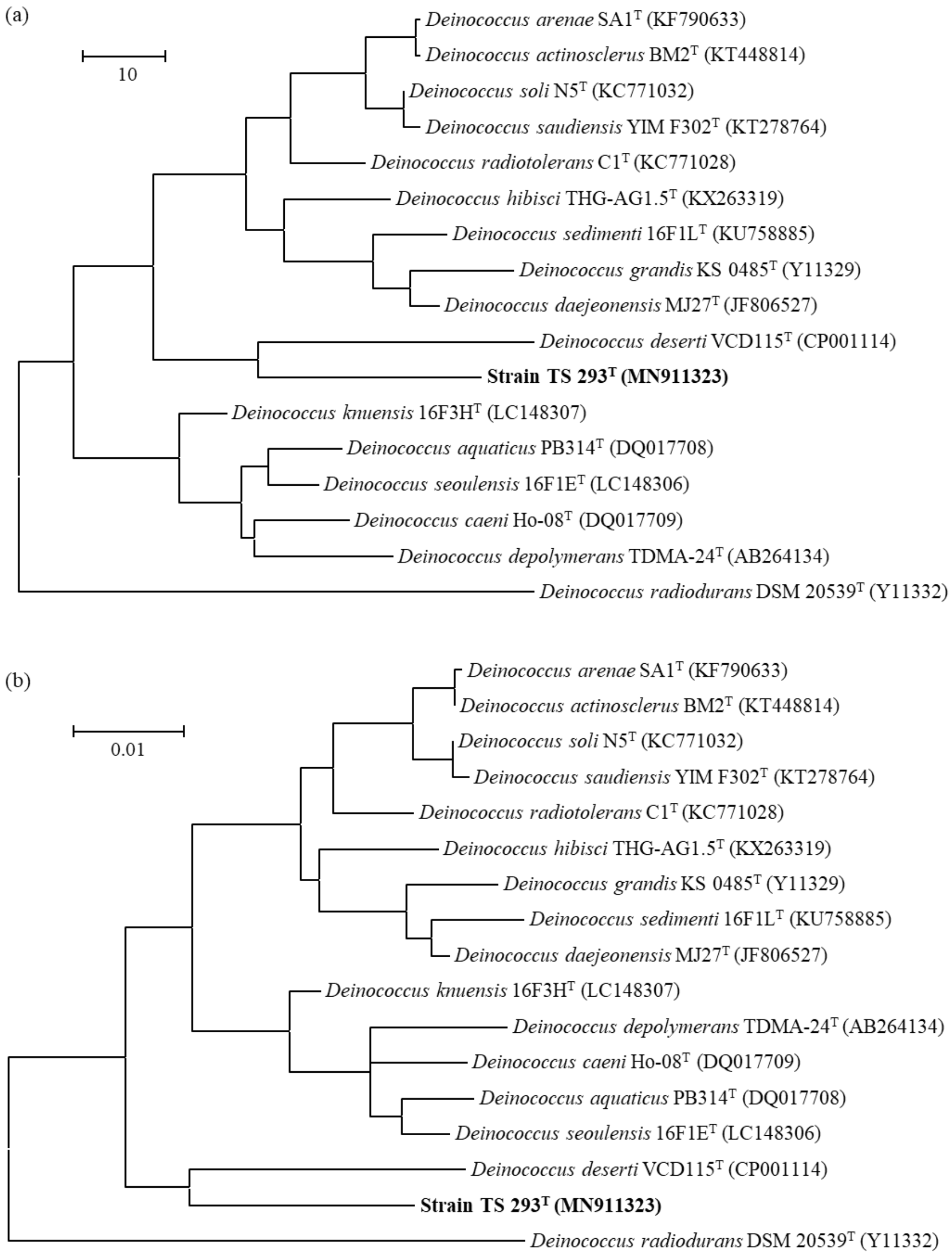

Supplementary Figure 1. Maximum-parsimony (a) and maximum-likelihood (b) trees based on 16S rRNA gene sequences showing the phylogenetic position of strain TS293^T.

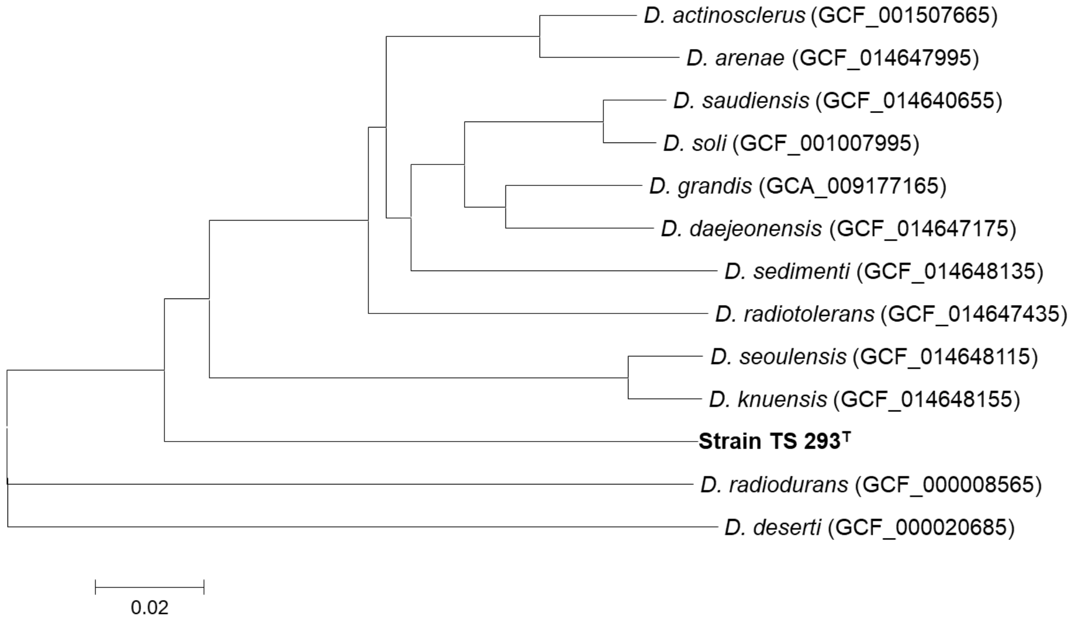

Supplementary Figure 2. Phylogenetic tree based on the whole-genome information. The evolutionary relatedness was examined by calculation of ANI between 13 sequenced *Deinococcs* species. Genome assembly records at NCBI are presented in parentheses. The bar reflects the normalized pairwise distance between genomes.

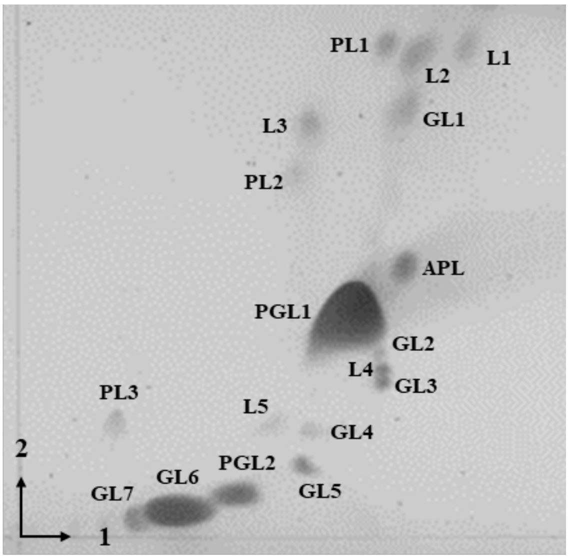

Supplementary Figure 3. Thin layer chromatograms of the polar lipids of TS293^T. PGL1-2, unidentified phosphoglycolipids; GL1-7, unidentified glycolipids; PL1-3, unidentified phospholipids; L1-5, unidentified polar lipids.
